# Persistence, adherence, healthcare resource utilization and costs for ocrelizumab in the real-world of the Campania Region of Italy

**DOI:** 10.1007/s00415-022-11320-7

**Published:** 2022-08-11

**Authors:** Marcello Moccia, Giuseppina Affinito, Giulia Berera, Giuseppina Marrazzo, Raffaele Piscitelli, Antonio Carotenuto, Maria Petracca, Roberta Lanzillo, Maria Triassi, Vincenzo Brescia Morra, Raffaele Palladino

**Affiliations:** 1 Multiple Sclerosis Clinical Care and Research Centre, Department of Neuroscience, Reproductive Science and Odontostomatology, University of Naples Federico II, via Sergio Pansini 5, 80131 Naples, Italy; 2 Department of Public Health, University of Naples Federico II, Naples, Italy; 3 ROCHE Spa, Viale GB Stucchi 110, 20900 Monza, MB Italy; 4 Hospital Pharmacy ASL NA1 Centro, Naples, Italy; 5 Department of Human Neurosciences, Sapienza University, Rome, Italy; 6 Department of Primary Care and Public Health, Imperial College, London, UK

**Keywords:** Multiple sclerosis, Ocrelizumab, Treatment, Persistence, adherence, Costs

## Abstract

**Aims:**

We aim to provide real-world evidence on the use of ocrelizumab for treating multiple sclerosis (MS), with specific regard to prescription pattern, adherence, persistence, healthcare resource utilization and related costs, also in relation to other disease-modifying treatments (DMTs).

**Methods:**

We included 2495 people with MS from the Campania Region (South Italy) who received first or switch DMT prescription from Jan 2018 to Dec 2020, and with at least 6-month follow-up. We collected hospital discharge records, drug prescriptions, and related costs, and calculated persistence (time from first prescription to discontinuation or switch to other DMT), adherence (proportion of days covered (PDC)), annualized hospitalization rate (AHR) for MS-related hospital admissions, and DMT costs.

**Results:**

Ocrelizumab was the most commonly prescribed DMT (*n* = 399; age = 45.74 ± 10.98 years; females = 224), after dimethyl fumarate (*n* = 588) and fingolimod (*n* = 401); 26% patients treated with ocrelizumab were naïve. When compared with ocrelizumab, the risk of discontinuation was higher for other highly active DMTs (HR = 3.78; *p* = 0.01), and low/medium efficacy DMTs (HR = 7.59; *p* < 0.01). When compared with ocrelizumab, PDC was similar to other highly active DMTs (Coeff = 0.01; *p* = 0.31), but higher for low/medium efficacy DMTs (Coeff = 0.09; *p* < 0.01). When compared with ocrelizumab, AHR was similar to other highly active DMTs (Coeff = 0.01; *p* = 0.51), and low/medium efficacy DMTs (Coeff = 0.01; *p* = 0.55). When compared with ocrelizumab, DMT monthly costs were higher for other highly active DMTs (Coeff = 92.30; *p* < 0.01), but lower for low/medium efficacy DMTs (Coeff = − 1043.61; *p* < 0.01).

**Discussion:**

Ocrelizumab was among the most frequently prescribed DMTs, with 26% prescriptions to treatment-naïve patients, suggesting its relevance in addressing unmet clinical needs (e.g., first approved treatment for primary progressive MS). Ocrelizumab was associated with the highest persistence, confirming its favorable benefit-risk profile. Costs for ocrelizumab were lower than those associated to similarly effective DMTs, in absence of changes in healthcare resource utilization.

## Introduction

Ocrelizumab is approved for the use in both relapsing–remitting and primary progressive multiple sclerosis (MS) [1, 2]. Ocrelizumab efficacy and safety have been preliminarily explored in clinical trials and their long-term extensions [3, 4]. More recently, insights on ocrelizumab real-world use and related clinical efficacy have been gained through clinical registries [5, 6]. However, clinical registries do not include healthcare resource utilization and, more in general, do not cover the complexity of MS management [7, 8]. Also, few studies have directly compared different DMTs in terms of efficacy measures [9]. Datasets based on routinely collected healthcare data can overcome these limitations and provide detailed information on healthcare resource utilization in the long term and on fully representative populations [10]. In the Campania Region of Italy, we have developed an algorithm, specific for individuals with a diagnosis of MS, to merge healthcare data (e.g. planned and unplanned hospital admissions with related diagnoses and costs) and prescription data [11], and to derive measures of DMT utilization (e.g., adherence, persistence) and economic viability.

Hereby, we aim to provide real-world evidence on the use of ocrelizumab, with specific regard to prescription pattern, persistence, adherence, healthcare resource utilization and related costs, and also to compare ocrelizumab to other DMTs, based on administration (e.g., injectable, oral, and infusion) and activity (e.g., low/medium efficacy and highly active DMTs).

## Methods

### Study design

This is a population-based study, based on the retrospective analysis of routinely collected healthcare data, prospectively recorded from 2018 to 2020, on individuals with a diagnosis of MS living in the Campania Region of Italy. The original dataset has been fully described elsewhere [11]. For the purposes of the present study, we have selected this time frame to include ocrelizumab-treated patients, from the beginning of its use in the real-world (first prescription is recorded on Nov 6, 2018).

The study was approved by the Federico II Ethics Committee (355/19). All patients signed informed consent authorizing the use of anonymized data collected routinely as part of the clinical practice, in line with data protection regulation (GDPR EU2016/679). The study was performed in accordance with good clinical practice and Declaration of Helsinki.

### Population

The dataset was created by merging different data sources of the Campania Region [11]. We specifically included all individuals resident in the Campania Region who had at least one MS record, from 2018 to 2020, in the Hospital Discharge Record database, the Regional Drug Prescription database, or the outpatient database with payment exemptions for MS. The case-finding algorithm has 99.0% sensitivity, with very low risk of missing individuals (2.7%) [11]. We have referred to both individual patients and individual treatment periods (ITPs), since the same patient could have been using different DMTs during the study period.

Inclusion criteria were: (1) new DMT prescriptions from Jan 1, 2018, to Dec 31, 2020 (switch from a previous DMT or DMT start in absence of previous treatment records, using data from 2015 to 2017 as characterization period); (2) DMT prescription maintained for at least 6 months (e.g., corresponding to two full infusions for ocrelizumab).

Exclusion criteria were: (1) individual treatment periods already including a DMT at baseline (Jan 1, 2018); (2) incomplete records; (3) lack of written consent to participate in the study; (4) residence outside of the Campania Region.

### Treatment variables

DMT prescriptions were collected, and following DMT groups were defined based on:

- DMT administration route: infusion (alemtuzumab, natalizumab), oral (cladribine, fingolimod, teriflunomide, dimethyl fumarate), and injection (glatiramer acetate, interferon beta-1a, interferon beta-1b, and peg-interferon beta-1a), using ocrelizumab as reference for comparison [12];
- DMT treatment line: low/medium efficacy (teriflunomide, dimethyl fumarate, glatiramer acetate, interferon beta-1a, interferon beta-1b, and peg-interferon beta-1a) and highly active treatments (alemtuzumab, natalizumab, cladribine, fingolimod), using ocrelizumab as reference for comparison [13, 14].

Based on DMT prescriptions in the previous 12 months, ITPs were classified into treatment naïve (no treatment records in the previous 12 months) and switcher patients (presence of previous treatment records).

### Persistence, adherence, healthcare resource utilization and costs

DMT discontinuation was defined as a switch to another DMT or complete discontinuation (i.e., no further record of medication initiation) [8, 13].

Adherence was calculated as the proportion of days covered (PDC) over 1-year time (total days covered during 1 year divided by 365 days of follow-up, using the expected refill/retreatment timing from current regulatory indications); PDC ≥ 0.8 was considered adherent [12]. Considering that some DMTs have low frequency administration that would have caused too much variability in estimating adherence in 6 months (e.g., alemtuzumab, cladribine, ocrelizumab), we have included in adherence analyses only patients with at least 12 months’ follow-up.

Healthcare resource utilization included MS-related and non-MS-related hospital admissions, that were classified based on the main discharge diagnosis. The number of hospital admissions was then reported on annual basis (annualized hospitalization rates (AHR)) [13, 15].

Direct healthcare costs were derived from regional datasets, referred to corresponding healthcare resource utilization, and inflated to the most recent values (2020), to avoid variations in price per unit of service through different years [13, 15].

We further collected age, sex, and, for patients with Hospital Discharge Records, Charlson Comorbidity Index [15, 16].

### Statistics

Study variables are presented as mean (± standard deviation), number (percent) or median (range), as appropriate.

Differences between DMT groups (using ocrelizumab as reference in the statistical models) were explored using Cox regression models (i.e., persistence), and linear regression models (i.e., adherence, AHR, costs), as appropriate. Covariates were age, sex, year of treatment start (2018, 2019, or 2020), treatment duration, and adherence; statistical models were then run for the subgroup of patients with hospital discharge records, also including Charlson comorbidity index among covariates.

Results were reported as adjusted coefficient (Coeff), adjusted hazard ratio (HR), 95% confidence intervals (95%CI), and *p* values, as appropriate. Statistical analyses were performed using Stata 15.0. Results were considered statistically significant for *p* < 0.05.

## Results

**Fig. 1:**
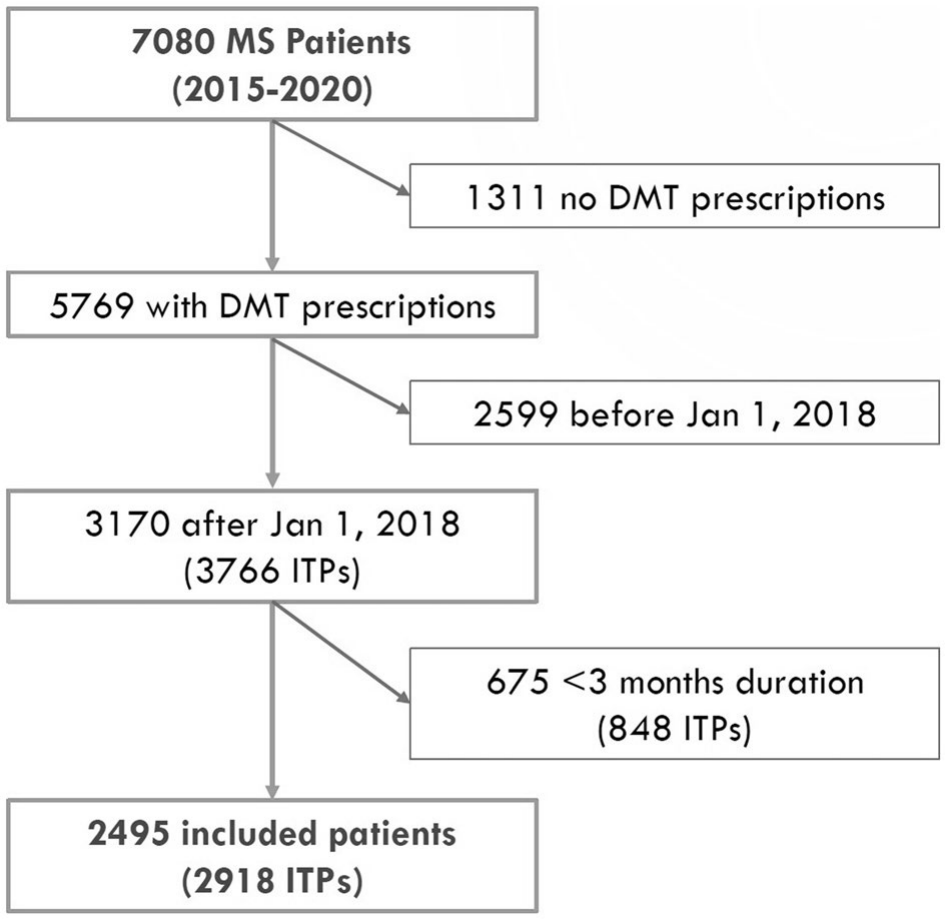
Study flow diagram. Figure shows the number of included and excluded patients, along with reasons for exclusion

From the population of people with MS in the Campania Region from 2015 to 2020 (*n* = 7080), we included 2495 individuals who were commenced on a DMT from 2018 to 2020, corresponding to 2918 ITPs (the same individual being treated with different DMTs within the study period). Reasons for exclusion are reported in Fig. 1. Demographics, comorbidities and treatment features of included patients (and respective ITPs) are reported in Table 1.

**Table 1:** Demographics, comorbidities and treatment features

DMT	Patients (*n*)	ITPs			Age (years)	Females (*n*)	Charlson Comorbidity Index			
		2018	2019	2020			0	1–2	3–4	≥ 5
Ocrelizumab	398	27	281	91	45.74 ± 10.98	224	395	5	–	–
Alemtuzumab	31	18	13	0	35.39 ± 8.31	21	31	–	–	–
Natalizumab	261	82	72	108	34.05 ± 10.99	183	360	2	–	–
Cladribine	30	0	26	4	43.13 ± 11.97	22	30	–	–	–
Fingolimod	399	197	139	65	39.17 ± 11.42	259	398	3	–	–
Teriflunomide	305	176	71	59	48.51 ± 11.18	202	302	4	–	–
Dimethyl fumarate	587	269	196	123	38.98 ± 12.10	408	583	3	1	–
Interferon beta1a im	87	63	14	10	48.84 ± 12.73	59	87	–	–	–
Interferon beta1b	67	52	14	7	52.12 ± .10.10	40	67	–	–	–
Glatiramer acetate	239	175	182	41	46.73 ± 11.66	164	236	3	–	–
Peg-interferon beta1a	80	39	28	13	39.86 ± 13.62	60	80	–	–	–
Interferon beta1a sc	262	179	48	36	40.88 ± 12.54	199	262	1	–	–

Overall, we included 398 patients treated with ocrelizumab, corresponding to 399 ITPs. Looking at administration route, we included 293 ITPs with other infusion DMTs (alemtuzumab, natalizumab), 1325 with oral DMTs (cladribine, fingolimod, teriflunomide, dimethyl fumarate), and 901 with injectable DMTs (glatiramer acetate, interferon beta-1a, interferon beta-1b, and peg-interferon beta-1a). Looking at efficacy line, we included 724 ITPs with other highly active DMTs (alemtuzumab, natalizumab, cladribine, fingolimod), and 1795 with low/medium efficacy DMTs (teriflunomide, dimethyl fumarate, interferon beta-1a, interferon beta-1b, and peg-interferon beta-1a). Most frequently prescribed DMTs were dimethyl fumarate (*n* = 588, 20.1%), fingolimod (*n* = 401, 13.7%) and ocrelizumab (*n* = 399, 13.6%), with ocrelizumab being the most frequently prescribed DMT in 2019. Also, we observed an overall drop in new DMT prescriptions in 2020 (Table 1).

Most patients treated with ocrelizumab were newly diagnosed and drug naïve (*n* = 104), followed by patients previously treated with fingolimod (*n* = 76), dimethyl fumarate (*n* = 54), teriflunomide (*n* = 51), glatiramer-acetate (*n* = 37), natalizumab (*n* = 34), interferon beta1a (*n* = 16), interferon beta1b (*n* = 13), alemtuzumab (*n* = 12), and peg-interferon beta1a (*n* = 2).

**Fig. 2:**
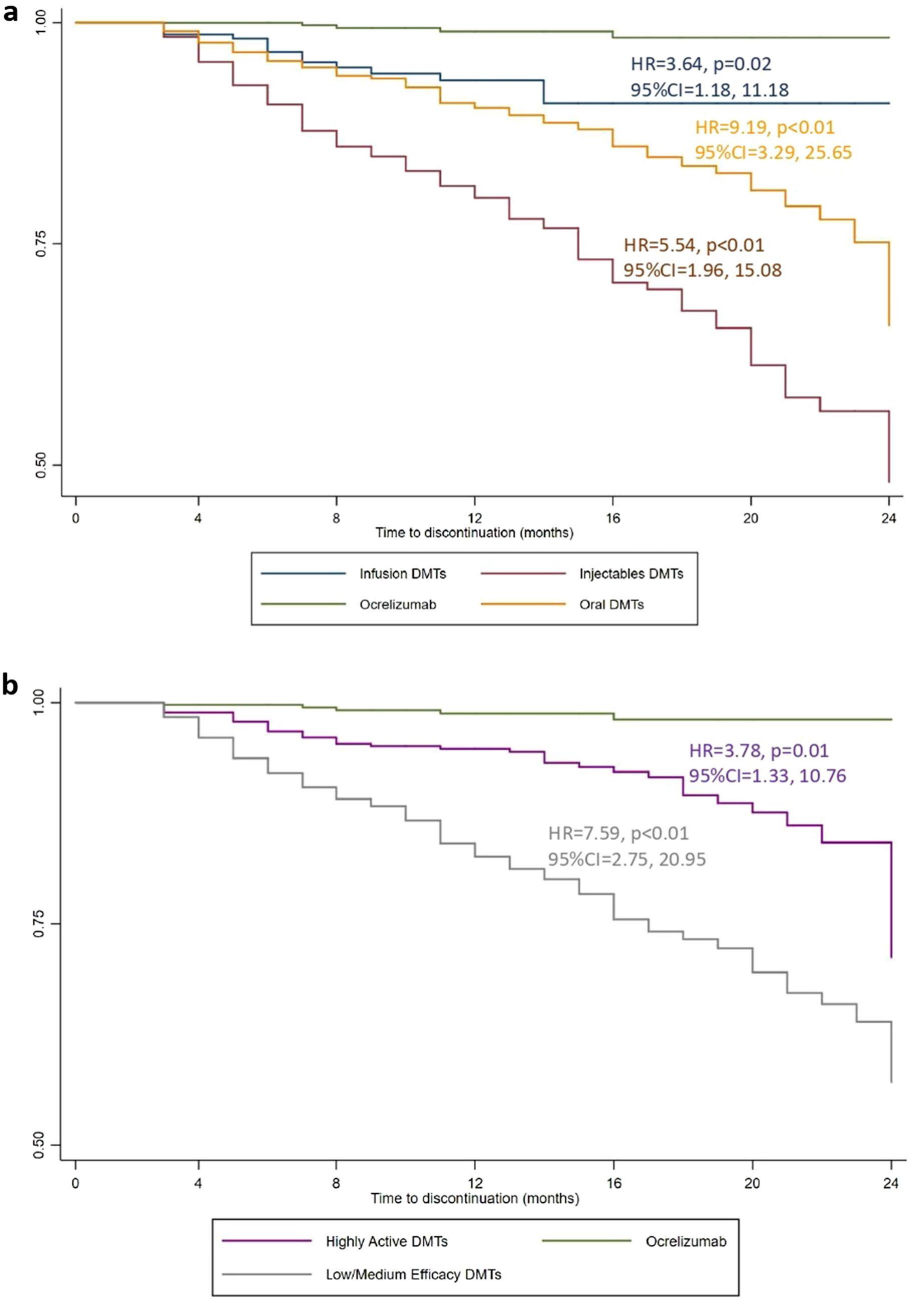
Kaplan–Meier estimates of treatment persistence. Adjusted hazard ratio (HR), coefficients (Coeff) and p-values are shown from Cox regression models evaluating administration route (**a**) and clinical efficacy (**b**), and including age, sex, year of treatment start (2018, 2019, or 2020), treatment duration, and adherence as covariates

ITP durations and number of patients switching to other DMT or completely discontinuing DMTs are reported in Table 2. A minority of ocrelizumab ITPs was discontinued (4 over 399), after 13.71 ± 5.42 months; in particular, 1 patient was switched to natalizumab, 2 patients to dimethyl fumarate, and 1 patient to interferon beta1a. When compared with ocrelizumab, the risk of discontinuation was higher for other infusion (HR = 3.64; 95%CI = 1.18, 11.18; *p* = 0.02), oral (HR = 9.19; 95%CI = 3.29, 25.65; *p* < 0.01) and injectable DMTs (HR = 5.54; 95%CI = 1.96, 15.08; *p* < 0.01) (Fig. 2a). Similarly, when compared with ocrelizumab, the risk of discontinuation was higher for other highly active (HR = 3.78; 95%CI = 1.33, 10.76; *p* = 0.01), and low/medium efficacy DMTs (HR = 7.59; 95%CI = 2.75, 20.95; *p* < 0.01) (Fig. 2b). Results were confirmed also after adjusting by Charlson Comorbidity index.

**Table 2:** Treatment duration

DMT	ITP duration (months)		Switch to other DMT	Complete DMT discontinuation
	Mean ± SD	Median (IQR)		
Ocrelizumab	13.71 ± 5.42	13 (8–19)	4	0
Alemtuzumab	13.77 ± 2.62	13 (12–15)	0	17
Natalizumab	15.53 ± 9.65	12 (7–24)	73	17
Cladribine	11.80 ± 3.21	13 (12–14)	0	0
Fingolimod	19.41 ± 9.97	19 (11–29)	135	62
Teriflunomide	19.79 ± 10.74	19 (10–30)	116	39
Dimethyl fumarate	19.10 ± 10.55	19 (10–29)	64	64
Interferon beta1a im	23.73 ± 12.03	30 (10–35)	130	17
Interferon beta1b	24.32 ± 12.32	30 (10–35)	93	24
Glatiramer acetate	23.41 ± 11.24	21 (9–34)	140	45
Peg-interferon beta1a	16.49 ± 10.38	15 (7–24)	85	16
Interferon beta1a sc	21.61 ± 11.50	23 (11–34)	248	56

Table shows mean (± standard deviation (SD)) and median (and interquartile range (IQR)) of duration of IPTs, and number of patients that were switched to other DMT or were completely discontinued from DMT

Adherence to treatment is reported in Table 3. When compared with ocrelizumab, adherence (PDC) was lower for oral DMTs (Coeff = − 0.18; 95%CI = − 0.26, − 0.12; *p* < 0.01), but similar to other infusion (Coeff = − 0.08; 95%CI = − 0.19, 0.02; *p* = 0.14), and injectable DMTs (Coeff = − 0.01; 95%CI = − 1.11, 0.11; *p* = 0.90). When compared with ocrelizumab, adherence was lower for other highly active DMTs (Coeff = − 0.11; 95%CI = − 0.19, − 0.02; *p* < 0.01), and low/medium efficacy DMTs (Coeff = − 0.18; 95%CI = − 0.26, − 0.10; *p* < 0.01). Results were confirmed also after adjusting by Charlson Comorbidity index.

**Table 3:** Adherence

DMT	PDC	PDC > 0.8	
Ocrelizumab	0.99 ± 0.24	155/177	88%
Alemtuzumab	1.02 ± 0.08	15/15	100%
Natalizumab	0.93 ± 0.17	41/51	80%
Cladribine	1.14 ± 0.37	7/7	100%
Fingolimod	0.89 ± 0.27	90/125	72%
Teriflunomide	1.09 ± 0.18	54/57	94%
Dimethyl fumarate	0.62 ± 0.53	50/114	43%
Interferon beta1a im	1.04 ± 0.35	5/7	71%
Interferon beta1b	0.83 ± 0.12	1 /2	50%
Glatiramer acetate	0.98 ± 0.27	9/11	81%
Peg-interferon beta1a	0.95 ± 0.28	11/18	61%
Interferon beta1a sc	1.01 ± 0.27	16/21	76%

Table shows the proportion of days covered (PDC) for each DMT, calculated as the total days covered during 1 year divided by 365 days of follow-up (as from current regulatory indications), for each ITP. The number and percent of patients with PDC above 80% is also reported

Healthcare resource utilization and costs are reported in Table 4. When compared with ocrelizumab, AHR was higher for other infusion DMTs (Coeff = 0.05; 95%CI = 0.01, 0.09; *p* = 0.03), and similar to oral (Coeff = − 0.01; 95%CI = − 0.03, 0.03; *p* = 0.97) and injectable DMTs (Coeff = 0.01; 95%CI = − 0.02, 0.05; *p* = 0.45). When compared with ocrelizumab, AHR was similar to other highly active (Coeff = 0.01; 95%CI = − 0.02, 0.04; *p* = 0.51), and low/medium efficacy DMTs (Coeff = 0.01; 95%CI = − 0.02, 0.04; *p* = 0.55). Results were confirmed also after adjusting by Charlson Comorbidity index.

**Table 4:** Healthcare resource utilization and costs

DMT	MS admissions			AHR	DMT costs
	Regular	Day hospital	Costs (EUR/month)		(EUR/month)
Ocrelizumab	17	397	83.01 ± 248.66	0.07 ± 0.37	1226.81 ± 219.62
Alemtuzumab	5	24	39.76 ± 54.13	0.28 ± 0.57	2272.26 ± 313.19
Natalizumab	14	267	93.34 ± 427.70	0.15 ± 0.83	1416.20 ± 272.77
Cladribine	0	8	15.14 ± 28.24	0.00 ± 0.00	1817.31 ± 821.72
Fingolimod	7	314	49.87 ± 78.97	0.01 ± 0.11	1465.45 ± 250.70
Teriflunomide	16	105	24.23 ± 58.44	0.06 ± 0.30	780.69 ± 126.49
Dimethyl fumarate	15	233	27.32 ± 64.36	0.02 ± 0.18	953.14 ± 161.20
Interferon beta1a im	1	13	5.64 ± 16.31	0.01 ± 0.06	763.40 ± 142.85
Interferon beta1b	2	4	16.52 ± 130.55	0.02 ± 0.11	445.10 ± 97.68
Glatiramer acetate	15	43	18.46 ± 76.49	0.08 ± 0.37	454.12 ± 161.62
Peg-interferon beta1a	1	7	9.81 ± 54.19	0.05 ± 0.44	878.93 ± 144.60
Interferon beta1a sc	7	91	16.12 ± 31.73	0.02 ± 0.16	797.34 ± 171.44

Table shows number of regular and day hospital MS-related admissions, and related costs. Annualized hospitalization rate (AHR) for MS-related admissions is also reported. Costs are based on actual DMT refill/administration per patient, and are referred to a month of 30.5 days

When compared with ocrelizumab, monthly costs for MS hospital admissions were similar to other infusion DMTs (Coeff = 7.83; 95%CI = − 12.94, 28.61; *p* = 0.46), but lower for oral (Coeff = − 18.95; 95%CI = − 35.27, − 2.64; *p* < 0.01) and injectable DMTs (Coeff = − 28.25; 95%CI = − 46.44, − 2.64; *p* = 0.02). When compared with ocrelizumab, monthly costs for MS hospital admissions were similar to other highly active (Coeff = − 0.77; 95%CI = − 18.12, 16.57; *p* = 0.93), but lower for low/medium efficacy DMTs (Coeff = − 26.02; 95%CI = − 42.45, − 9.58; *p* < 0.01). Results were confirmed also after adjusting by Charlson Comorbidity index.

When compared with ocrelizumab, monthly costs were similar to other infusion DMTs (Coeff = − 57.28; 95%CI = − 119.15, 4.59; *p* = 0.07), but lower for oral (Coeff = − 675.83; 95%CI = − 723.16, − 628.50; *p* < 0.01) and injectable DMTs (Coeff = − 675.83; 95%CI = − 1205.92, − 1100.75; *p* < 0.01). When compared with ocrelizumab, monthly costs were higher for other highly active DMTs (Coeff = 92.30; 95%CI = 53.01, 131.60; *p* < 0.01), but lower for low/medium efficacy DMTs (Coeff = − 1043.61; 95%CI = − 1080.02, − 1007.20; *p* < 0.01). Results were confirmed also after adjusting by Charlson Comorbidity index.

## Discussion

In this population-based study, we specifically aimed to describe the use of ocrelizumab in the real-world of the Campania Region of Italy, with regards to prescription pattern, persistence, adherence, healthcare resource utilization and related costs. Ocrelizumab was the most frequently prescribed DMT for MS in 2019, with 26% prescriptions being made to treatment-naïve MS patients, suggesting it was addressing unmet needs in the MS treatment scenario. This is the first real-world study on ocrelizumab describing both utilization pattern (i.e., persistence, adherence), and related healthcare resource utilization and costs.

When compared with other high-efficacy DMTs, ocrelizumab was used on much more complex populations (i.e., older age, higher comorbidity burden), as already described by some previous studies [17–19]. Notwithstanding this, in our cohort, only 1% patients were discontinued from ocrelizumab, suggesting optimal efficacy and safety [20, 21], with higher persistence rates compared with other oral, infusion and injectable DMTs. This could be at least in part due to the use of ocrelizumab on newly diagnosed and treatment-naïve patients in our cohort, which is a known factor of optimal treatment response [5]. Ocrelizumab has already proved high persistence rates in previous studies [12, 19, 22, 23], with efficacy and safety issues being the most common causes of discontinuation [19, 23]. Of note, relapses, disability progression and MRI activity are expected to occur in a minority of patients treated with ocrelizumab [17, 18, 24]. Taken together, our data suggest that ocrelizumab high persistence rates might be a consequence of optimal efficacy and safety.

We also found high rates of adherence to ocrelizumab compared with lower and similar efficacy class. While we have to acknowledge that adherence analyses were run on the subset of patients with at least 12 months of follow-up, our rate of adherence is in line with previous similar studies [12, 25], and overall suggests optimal safety profile (e.g., no need to delay infusions). Looking at previous real-world studies, side effects were reported by 10% of patients, mostly consisting of mild infusion-related reactions and infections [18], independently from age [17].

The main novelty of our study is the inclusion of healthcare resource utilization and costs. In particular, ocrelizumab was associated with lower direct treatment costs, but was associated with similar probability of MS-related hospital admissions and costs, when compared with other DMTs similar in administration route (e.g., natalizumab, alemtuzumab) and efficacy class (e.g., natalizumab, alemtuzumab, cladribine, fingolimod). Similarly, in a previous US claims’ study including 189 patients treated with ocrelizumab, alemtuzumab or natalizumab for 1 year, authors showed reduced costs for ocrelizumab treatment and related procedures [26]. Overall, these findings suggest that ocrelizumab is less expensive but similarly effective to other high-efficacy DMTs.

Limitations of our study include the generalizability of our results, since we only included patients from a specific Italian region. However, our cohort had similar distribution (e.g., age, DMT use), when compared to other international studies [17, 18, 27], and, hence, may reflect the general MS population treated with ocrelizumab. Also, rates of disability progression, relapses and related healthcare resource utilization are expected to increase over the follow-up [28]. Therefore, longer follow-up is warranted to confirm our findings. In addition, we have also included 2020 year in the analysis, with the bias of COVID19 pandemic that could have caused extended interval dosing for ocrelizumab [29]; however, based on adherence results, this was not the case for most infusions and the drop of new prescriptions in 2020 is in line with a previous English study [30]. Our study also holds limitations derived from the use of routinely collected healthcare data, including the definition of MS-related hospital admission based on the primary diagnosis that could be biased by the physician perspective. We compared ocrelizumab to other approved DMTs specifically approved for MS, while did not extend the analysis to other treatments (e.g., rituximab) due to sample size constraints and possible selection bias, deriving from their use in highly selected populations (e.g., non-responders to approved DMTs).

In conclusion, we confirmed previous results on high persistence and adherence rates of ocrelizumab, when compared with DMTs of similar efficacy and mode of administration. We also showed that ocrelizumab is less expensive than other high-efficacy DMTs, while possibly equally effective based on indirect measures on routinely collected healthcare data (i.e., AHR and related costs).
